# Diagnosis, treatment, and prognosis of adult pancreatoblastoma

**DOI:** 10.1002/cam4.70132

**Published:** 2024-08-20

**Authors:** Jiaqian Yuan, Yong Guo, Yan Li

**Affiliations:** ^1^ The First Clinical Medical College Zhejiang Chinese Medical University Hangzhou China; ^2^ Department of Medical Oncology The First Affiliated Hospital of Zhejiang Chinese Medical University Hangzhou China

**Keywords:** adult pancreatoblastoma, *APC*, pancreatic cancer, squamous corpuscles, survival analysis

## Abstract

**Background:**

Pancreatoblastoma (PB) is one of the rare malignant tumors that typically occurs in children. Cases of PB in adults are highly unusual. This disease often presents with subtle symptoms and lacks characteristic clinical manifestations, leading to diagnostic challenges.

**Objective:**

This study integrates the relevant literature on adult PB, conducting data analysis on clinical features, laboratory and imaging results, pathological characteristics, and treatments according to inclusion and exclusion criteria. Kaplan–Meier univariate analysis and Log‐rank tests are employed to analyze survival data from adult PB follow‐up, exploring factors influencing prognosis.

**Results:**

A total of 65 articles were included, encompassing 103 cases of adult PB. The average age of PB patients was 41.78 years (range 19–81 years), and the male‐to‐female ratio was 1.06:1. Patients frequently presented with abdominal pain as the initial symptom. Laboratory results lacked specificity and imaging findings often presented as large, well‐defined masses. PB exhibited distinctive pathological features, including squamous corpuscles (*n* = 76, 89.41%) and acinar differentiation (*n* = 34, 40%), with frequent positive expression of Trypsin, Chymotrypsin, and AACT (Alpha‐1‐Antichymotrypsin). APC (Adenomatous Polyposis Coli) gene mutation was the most common molecular alteration in adult PB. During the follow‐up period, 43.59% of patients died (range 3 days to 348 months). The primary factors affecting prognosis were the presence of metastasis (*χ* ^2^ = 3.996, *p* = 0.046) and incomplete surgical resection (*χ* ^2^ = 5.586, *p* = 0.018), with mean survival times of 48 months and 27 months, respectively.

**Conclusions:**

PB in adults is an invasive tumor. The key to distinguishing PB from other pancreatic tumors lies in recognizing its unique pathological feature, the squamous corpuscles. Timely and complete surgical resection is the preferred treatment following diagnosis. Patients with incomplete resection or the presence of lymph nodes or (and) distant metastases have a poor prognosis.

## 1 INTRODUCTION

Pancreatoblastoma (PB) is a rare malignant pancreatic epithelial tumor, accounting for less than 1% of all types of pancreatic tumors.^1^ Horie et al.^2^ first proposed the concept of PB in 1977. Due to its prevalence in children, constituting 25% of tumors in children under 10 years old, it was previously often referred to as “infantile pancreatic carcinoma.” It was not until 1986 that Palosaari et al.^3^ reported the first case of adult PB. Adult cases are typically sporadic, though they have rarely been reported in patients with familial adenomatous polyposis (FAP), and the clinical characteristics and treatments of PB in adults differ from those in children.^1^ The exact pathogenic mechanism of adult PB remains unclear and may be associated with certain gene mutations. Adult PB tends to be more invasive, and compared to children, adults with this malignant disease have a worse prognosis.^1^ Due to the lack of specific symptoms and the rarity of this disease, PB is easily overlooked and misdiagnosed in the clinic, which may lead to a delay in the diagnosis and further shorten the patient's survival. PB is sporadically reported in the literature, with case reports being predominant and a lack of systematic analysis. Therefore, this study integrates existing literature to systematically summarize and analyze PB based on clinical features, laboratory and imaging results, pathological characteristics, treatments, and survival analysis, aiming to strengthen the understanding of this disease and provide guidance for its clinical recognition and management.

## 2 METHODS

We searched Pubmed, Embase, Web of Science, and CNKI from inception to March 20, 2024. The search terms used were “pancreatoblastoma” and “adult pancreatoblastoma.” Additionally, relevant journals, bibliographies, and reviews were manually searched for additional articles. The inclusion criteria were: (1) authentic clinical case reports; (2) adult primary PB patients (over 18 years old) with definite pathological diagnosis. The exclusion criteria were: (1) duplicate literature; (2) non‐adult or non‐primary PB patients; (3) fundamental experiment study; (4) unavailable reviews; (5) largely missing clinical data.

**FIGURE 1:**
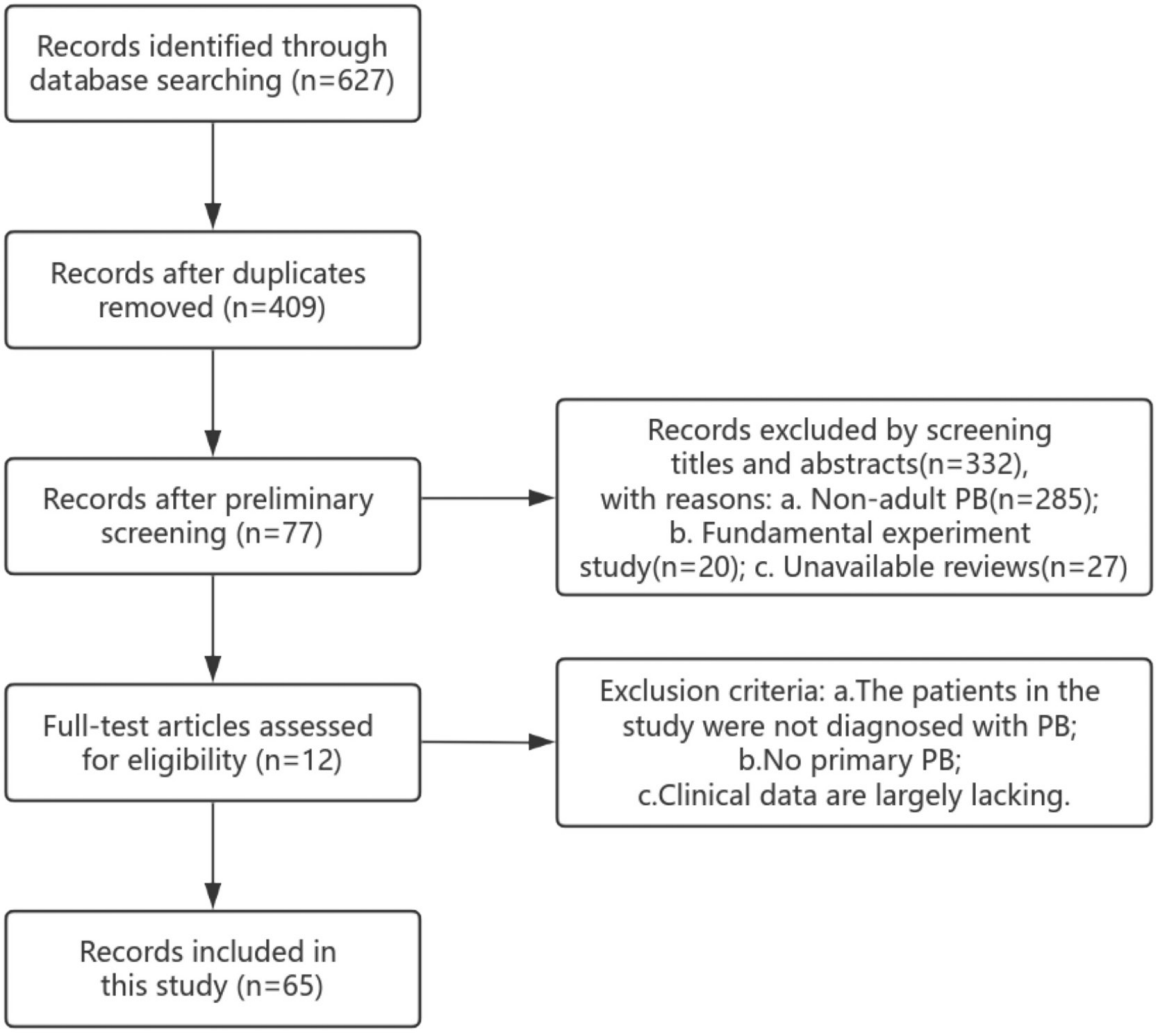
Flow diagram.

After screening, 65 references were identified (Figure 1). We conducted a detailed review of the literature, reviewing the relevant cases and extracting information, including the general condition of PB patients (sex, age, symptoms), laboratory and imaging findings at initial presentation, pathological characteristics (immunohistochemical and molecular pathology) and treatment methods for summary statistics. In addition, survival and prognostic analyses were performed for the disease.

## 3 RESULTS

### 3.1 Epidemiology and clinical Features

A total of 65 literature articles were included in this study,^3^, ^4^, ^5^, ^6^, ^7^, ^8^, ^9^, ^10^, ^11^, ^12^, ^13^, ^14^, ^15^, ^16^, ^17^, ^18^, ^19^, ^20^, ^21^, ^22^, ^23^, ^24^, ^25^, ^26^, ^27^, ^28^, ^29^, ^30^, ^31^, ^32^, ^33^, ^34^, ^35^, ^36^, ^37^, ^38^, ^39^, ^40^, ^41^, ^42^, ^43^, ^44^, ^45^, ^46^, ^47^, ^48^, ^49^, ^50^, ^51^, ^52^, ^53^, ^54^, ^55^, ^56^, ^57^, ^58^, ^59^, ^60^, ^61^, ^62^, ^63^, ^64^, ^65^, ^66^, ^67^ totaling 103 adult patients with PB. Of these, 53 (51.46%) were male and 50 (48.54%) were female, with a male‐to‐female ratio of 1.06:1. The age of the patients ranged from 19 to 81 years old, with 58 cases (56.31%) aged 19–44 years old, 20 cases (19.42%) aged 45–59 years old, and 25 cases (24.27%) aged older than 60. The mean age were 41.78 years old. The analysis revealed that PB predominated among younger age groups.

Among the primary symptoms reported by patients during their initial consultation (mentioned in 85 cases), the most common complaint was abdominal pain, reported in 40 cases (47.06%). This was followed by weight loss in 19 cases (22.35%), abdominal masses in 16 cases (18.82%), and jaundice in 15 cases (17.65%). Additional symptoms mentioned more than three times included abdominal discomfort (11.76%), diarrhea (7.06%), and nausea (4.71%) (Table 1). Only 25 cases included information on the patients' medical history. Among these, seven cases (28%) explicitly stated no significant medical history, and three cases (12%) had FAP.

**TABLE 1:** Clinical features.^a^

Symptom	*n* (%)	Symptom	*n* (%)
Abdominal pain	40 (47.06)	Chest pain	2 (2.35)
Weight loss	19 (22.35)	Melena	2 (2.35)
Abdominal mass	16 (18.82)	Fatigue	2 (2.35)
Jaundice	15 (17.65)	Anorexia	2 (2.35)
Abdominal discomfort	10 (11.76)	Dysphagia	1 (1.18)
Diarrhea	6 (7.06)	Polyarthralgia	1 (1.18)
Nausea	4 (4.71)	Exophthalmos	1 (1.18)
Vomit	3 (3.53)	Tachypnea	1 (1.18)
Altered bowel habit	3 (3.53)	Ascites	1 (1.18)

^a^ *n*, number; percentage accurate to 2 decimal places.

Furthermore, this study conducted a statistical analysis of the metastatic status of PB patients at the time of their initial diagnosis (including imaging and pathological evidence). There were 58 cases (56.31%) without metastasis and 45 cases (43.69%) with metastasis, including 9 cases (20%) of lymph node metastasis. Among patients with distant metastases, liver metastasis was the most common, accounting for 34 cases (75.56%), with 28 cases of liver metastasis alone. This is followed by lung metastasis in three cases (6.67%), although not as a sole site. There were seven cases (15.56%) involving metastases to two or more organs, and among these, liver metastasis combined with other metastases accounted for six cases (13.33%) (Table 2).

**TABLE 2:** The condition of metastasis.

Metastasis	Organ(s) involved	*n* (%)
Lymph node	–	9 (20.00)
Single‐organ	Liver	28 (62.22)
	Enterocoelia	1 (2.22)
Two‐organ	Liver, duodenum	2 (4.44)
	Kidney, adrenal gland	1 (2.22)
	Liver, lung	1 (2.22)
	Liver, peritoneum	1 (2.22)
Three or more	Liver, lung, breast	1 (2.22)
	Liver, lung, kidney, adrenal gland, spleen	1 (2.22)

### 3.2 Laboratory and imaging findings

A total of 53 cases documented laboratory examination results, of which 28 (52.83%) showed abnormal results. Elevated levels of tumor markers were detected in 24 cases (45.28%), including AFP (Alpha‐Fetoprotein) (*n* = 9, 16.98%), CA19‐9 (Carbohydrate Antigen 19–9) (*n* = 7, 13.21%), CA125 (*n* = 3, 5.66%), NSE (Neuron‐Specific Enolase) (*n* = 3, 5.66%), CEA (Carcinoembryonic Antigen) (*n* = 1, 1.89%), and CgA (Chromogranin A) (*n* = 1, 1.89%). Additionally, eight cases (15.09%) showed elevated liver enzymes, and six cases (11.32%) demonstrated increased bilirubin levels. Other abnormalities included elevated lipase and β2‐microglobulin levels, decreased white blood cell count, and electrolyte imbalances, as specified in Table 2.

Imaging findings were reported in 99 cases. The specific site of primary tumor invasion was noted in 89 cases, with the head of the pancreas being the most common site in 41 cases (46.07%), followed by the tail of the pancreas in 33 cases (37.08%) and the body of the pancreas in 8 cases (8.99%). Tumor size was mentioned in 80 cases, with imaging including MRI (magnetic resonance imaging) in 2 cases, ultrasound in 3 cases, and CT (computed tomography) in the remainder. The maximum diameter of the tumor ranged from 1.2 to 21.5 cm. The mean of maximum diameter was 7.39 cm; 45 cases (56.25%) had tumors larger than 5 cm. Initial imaging revealed metastasis in 38 out of 95 cases (40%), including five patients (5.26%) with only lymph node metastasis and 33 (34.74%) with distant metastasis (Table 3).

**TABLE 3:** The results of laboratory and imaging^a^.

	Item	*n* (%)	Item	*n* (%)
Laboratory	Normal	25 (47.17)	NSE	3 (5.66)
	AFP	9 (16.98)	Electrolyte disturbance	2 (3.77)
	Liver enzymes	8 (15.09)	CEA	1 (1.89)
	CA19‐9	7 (13.21)	CgA	1 (1.89)
	Bilirubin	6 (11.32)	WBC	1 (1.89)
	HGB	4 (7.55)	Lipase	1 (1.89)
	CA125	3 (5.66)	β2‐microglobulin	1 (1.89)

		Characteristic	*n* (%)	Characteristic		*n* (%)
Imaging	Site	Head	41 (46.07)	Size (cm)	<5	35 (43.75)
		Tail	33 (37.08)		≥5, <10	26 (32.50)
		Body	8 (8.99)		≥10, <15	12 (15.00)
		Body and tail	2 (2.25)		≥15, <20	6 (7.50)
		Neck	2 (2.25)		≥20	1 (1.25)
		Ampulla Vateri	2 (2.25)	Metastasis^b^	LN	5 (5.26)
		Retroperitoneal	1 (1.12)		Distant	33 (34.74)

^a^ LN, lymph node; Percentage accurate to 2 decimal places.

^b^ Only metastases that mention imaging findings in all the literature are counted here; LN, Number of cases with simple lymph node metastasis without distant metastasis.

Adult primary PB lacks characteristic imaging findings. According to statistics, CT findings often reveal large, well‐defined masses with regular morphology and clear margins (*n* = 19). When the tumor infiltrates adjacent organs or tissues, it may present with indistinct margins (*n* = 11). The average maximum diameter is 7.39 cm, with most tumors located in the pancreatic head or tail, typically appearing as solid‐cystic lesions (*n* = 16) and predominantly hypodense (*n* = 8). Necrosis (*n* = 8) or calcifications (*n* = 3) may be observed within some masses. Post‐contrast enhancement may show heterogeneous enhancement of the tumor (*n* = 5), with visible, mostly intact enhancing capsules (*n* = 9). Some masses may encase vessels such as the common bile duct (CBD), superior mesenteric artery (SMA), superior mesenteric vein (SMV), or portal vein (PV) (*n* = 6), or invade and compress adjacent organs (*n* = 16) such as the duodenum, stomach, or small intestine. Additionally, imaging in several cases demonstrates biliary or pancreatic duct obstruction and dilation due to mass growth (*n* = 14). Ultrasound typically reveals solid‐cystic hypoechoic masses (*n* = 7) with rich internal vascularity (*n* = 4).

### 3.3 Pathological characteristics

#### 3.3.1 Histology

Among the 103 cases of PB included, specific histopathological descriptions of the primary lesions were available for 85 cases, which underwent surgical resection or fine‐needle aspiration (FNA) sampling. In summary, tumor cells can be arranged into island‐like and geographic structures at the histological level under low magnification. Microscopically, the tumor tissue predominantly comprised epithelial and mesenchymal components (*n* = 12, 14.12%), with tumor cells often separated by dense fibrous bands (*n* = 14, 16.47%) and some cells dispersed within a necrotic background (*n* = 7, 8.24%). Many PB exhibited acinar differentiation (*n* = 34, 40%), characterized by relatively uniform cell composition, with possible ductal components and rare allogenic interstitial components, including tumor osteoid and cartilage (*n* = 3, 3.53%). Tumor cell morphology appeared regular or polygonal, showing polarity, with some cells displaying prominent nucleoli (*n* = 8, 9.41%) and regular (*n* = 10, 11.76%) nuclei of varying sizes with different degrees of nuclear atypia (*n* = 6, 7.06%). Nuclear division phenomena were occasionally observed (*n* = 7, 8.24%). Notably, the characteristic pathological manifestation of PB is squamous corpuscles composed of cellular nests (*n* = 76, 89.41%). These squamous corpuscles varied in density and distribution in different cases or different regions of the same case, comprising loosely aggregated larger spindle‐shaped cells, often originating from the center of small lobules and extending peripherally, forming fascicular and vague whirlpool‐like structures. Some demonstrated evident squamous epithelial differentiation, with cytoplasmic eosinophilic and transparent, and keratinization was observed in a few cases (*n* = 2, 2.35%). A subset of tumor cells exhibited a high mitotic rate (*n* = 17, 20%), with a small proportion demonstrating infiltration around lymphatic, vascular, or neural structures (*n* = 7, 8.24%) (Table 4).

**TABLE 4:** Pathological features^a^.

Characteristic	*n* (%)
Squamous corpuscles	76 (89.41)
Acinar differentiation	34 (40.00)
High mitosis rate	17 (20.00)
Fibrous bands	14 (16.47)
Epithelial and mesenchymal components	12 (14.12)
Nucleus	
Regular	10 (11.76)
Prominent	8 (9.41)
Division	7 (8.24)
Atypia	6 (7.06)
Necrosis	7 (8.24)
Infiltration	7 (8.24)
Osteoid and cartilage	3 (3.53)
Keratinization	2 (2.35)

^a^ All counts reflected in this table are based on cases with clear histopathological descriptions. Cases with ambiguous or indeterminate descriptions were systematically excluded to ensure the accuracy and specificity of the reported pathological data. Percentage accurate to 2 decimal places.

#### 3.3.2 Immunohistochemistry

The immunohistochemical (IHC) findings of 68 patients from all the literature were described in detail. Statistical analysis revealed that PB often expressed neuroendocrine markers, particularly Syn and CgA, with frequencies of 48 (70.59%) and 38 (55.88%), respectively. Additional markers included CD56 (Cluster of Differentiation 56), NSE, INSM1 (Insulinoma‐Associated 1), S‐100 (S‐100 Protein), and CD99. A total of 111 positive occurrences were recorded for this type of marker, involving 56 patients (82.35%). Epithelial markers were expressed in 41 patients (60.29%) with a total of 69 occurrences, including AE1/AE3 (Cytokeratin AE1/AE3), CK (Cytokeratin), CK5/6, 7, 8/18, 19 and CAM5.2 (Cytokeratin CAM5.2), EMA (Epithelial Membrane Antigen). Besides, acinar markers were also frequently positive, with Trypsin showing the most prominent expression in 31cases (45.59%), followed by Chymotrypsin and BCL‐10 (B‐Cell Lymphoma/Leukemia 10). β‐Catenin was often abnormally expressed in the nucleus and cytoplasm, within squamous corpuscles. In addition, expressions of AACT (Alpha‐1‐Antichymotrypsin), CD10, and CDX2 (Caudal Type Homeobox 2) were noted. Patients with elevated blood tumor markers (AFP and CA19‐9) often exhibited positive results in IHC. Ki‐67 was expressed in 26 patients, with 14 cases showing results greater than 30%, and the average value was 32.42% (Table 5).

**TABLE 5:** The positive signals of IHC^a^.

Signals		*n* (%)	Signals		*n* (%)
Epithelial markers	AE1/AE3	20 (29.41)	Neuroendocrine markers	Syn	48 (70.59)
	CK	13 (19.12)		CgA	38 (55.88)
	CK19	8 (11.76)		CD56	13 (19.12)
	CAM5.2	7 (10.29)		NSE	7 (10.29)
	CK8/18	6 (8.82)		INSM1	3 (4.41)
	CK7	5 (7.35)		S‐100	1 (1.47)
	CK5/6	5 (7.35)		CD99	1 (1.47)
	EMA	5 (7.35)	β‐Catenin		31 (45.59)
Acinar markers	Trypsin	31 (45.59)	AACT		14 (20.59)
	Chymotrypsin	18 (26.47)	AFP		5 (7.35)
	BCL‐10	5 (7.35)	CD10		4 (5.88)
Vimentin		3 (4.41)	CA19‐9		3 (4.41)
CDX2		3 (4.41)	Lipase		1 (1.47)
PCNA		1 (1.47)	Ki‐67^b^		32.42%

^a^ Percentage accurate to 2 decimal places.

^b^ The result of Ki‐67 is specific average.

#### 3.3.3 Molecular pathology

The genetic pathology of PB patients was studied in 14 of the included cases, with detailed results reported. APC (Alterations in the adenomatous polyposis coli)/β‐Catenin pathway were identified as crucial mechanisms in PB development (*n* = 10, 71.43%), with five cases showing mutations in the CTNNB1 (β‐Catenin) gene (*n* = 5, 35.71%). Normally, APC functions to inhibit the activity of β‐Catenin (encoded by the CTNNB1 gene), helping maintain the stability of intracellular β‐Catenin. However, mutations in the APC gene lead to abnormal accumulation and activation of β‐Catenin in the cell nucleus, consequently impacting the WNT (Wingless‐related integration site) signaling pathway. Notably, one patient with an APC gene mutation also harbored a SMAD4 (SMAD family member 4) gene mutation, while another patient with a SMAD4 gene mutation (*n* = 2, 14.29%) concurrently presented with a MEN1 (Multiple Endocrine Neoplasia Type 1) mutation (*n* = 1, 7.14%). SMAD4 gene mutations may disrupt the normal functioning of the TGF‐β signaling pathway, causing cells to lose their growth‐inhibitory response and thereby promoting tumor development. Furthermore, mutations in the RB1 (Retinoblastoma 1) gene, TSC2 (Tuberous Sclerosis Complex 2) gene, and FGFR2‐INA (Fibroblast Growth Factor Receptor 2 Intronic Non‐Coding RNA) fusion were each observed in one case (7.14%), all of which are closely associated with tumorigenesis.

### 3.4 Misdiagnose

The included medical records found that a total of 26 cases were misdiagnosed at the time of initial diagnosis. The most frequently mentioned misdiagnosis was pancreatic neuroendocrine neoplasms (PanNENs), with 13 cases (50%), of which 3 cases (11.54%) were specified as poorly differentiated. This was followed by solid pseudopapillary neoplasm of the pancreas in six cases (23.08%), acinar cell carcinomas in four cases (15.38%), gastrointestinal stromal tumor (GIST) in two cases (7.69%), and carcinoid in one case (3.85%).

### 3.5 Treatment and prognosis

#### 3.5.1 Treatment

**FIGURE 2:**
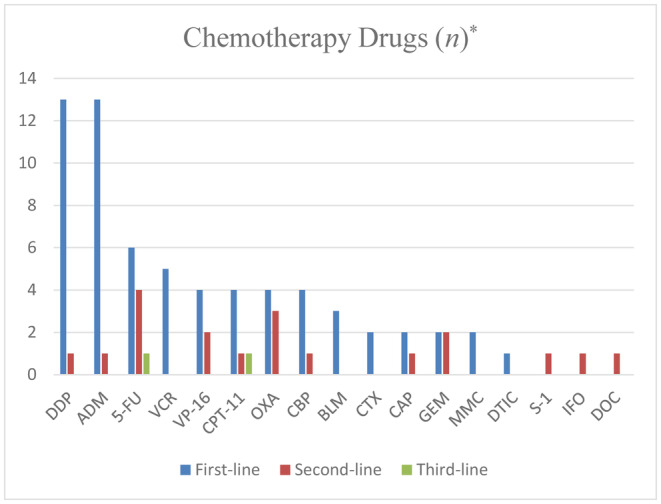
Chemotherapy drugs used at different stages. *VP‐16, etoposide; CTX, cyclophosphamide; CAP, capecitabine; GEM, gemcitabine; MMC, mitomycin; DTIC, dacarbazine; S‐1, tegafur; IFO, ifosfamide; DOC, docetaxel.

A total of 91 cases mentioned the treatment modalities post‐diagnosis of PB. The most frequently reported intervention was surgery (*n* = 78, 85.71%). Chemotherapy followed, with 34 individuals (37.36%), including two cases (2.20%) of neoadjuvant combined with postoperative adjuvant therapy, utilizing FOLFIRINOX (5‐fluorocrail+leucovorin+Irinotecan+Oxaliplatin) (5‐FU+LV+CPT‐11+OXA) and a single‐agent regimen of ADM (Adriamycin), respectively. Among the 27 patients (seven unknown) where chemotherapy drugs for adjuvant therapy were specified, the most commonly mentioned drugs were DDP (Cisplatin) and ADM, with their combination being the most frequent chemotherapy regimen (*n* = 7, 20.59%). Additionally, a combination of another platinum‐based drug (CBP, Carboplatin) with ADM was recorded twice (5.88%). Furthermore, FOLFIRINOX (*n* = 4, 11.76%) and PVB (Cisplatin +Vincristine +Bleomycin) (DDP+VCR+BLM) (*n* = 3, 8.82%) regimens were also relatively common. Second‐line therapy often involves chemotherapy regimens based on 5‐FU. For third‐line medication, one case explicitly stated the use of FOLFIRI (5‐FU+LV+CPT‐11)+BEV (bevacizumab) (Figure 2). Apart from BEV, the targeted drug Nintedanib (*n* = 2, 2.20%) was also mentioned. Immunologic drugs (*n* = 4, 4.40%) were similarly used in posterior line therapy, including Pembrolizumab, Ipilimumab, and Nivolumab, as mentioned in the literature. Ten patients (10.99%) had undergone radiotherapy, all having received chemotherapy previously or combined chemo‐radiotherapy. Four patients (4.40%) underwent liver interventional therapy due to liver metastasis, including transcatheter arterial chemoembolization (TACE) and ablation therapy. Two cases (2.20%) required palliative care due to delayed treatment. Other treatment modalities included autologous stem cell transplantation (ASCT), accounting for two cases (2.20%). Besides surgical intervention, chemotherapy, radiotherapy, targeted therapy, and immunotherapy were employed in treating PB.

#### 3.5.2 Prognosis

Survival data were available for 78 of the 103 cases, with a follow‐up period ranging from 3 days to 348 months. Among them, 14 patients (17.95%) survived for 5 years or more, and 4 (5.13%) for over 10 years. Of these cases, 34 patients (43.59%) died during the follow‐up period, with an average survival period of 22.84 months and a median survival time of 34 months. Fifteen of the 30 patients (50%) with adjuvant chemotherapy for whom survival data were provided died during the follow‐up period, and 6 of the 9 patients (66.67%) who had combined radiotherapy died.

**FIGURE 3:**
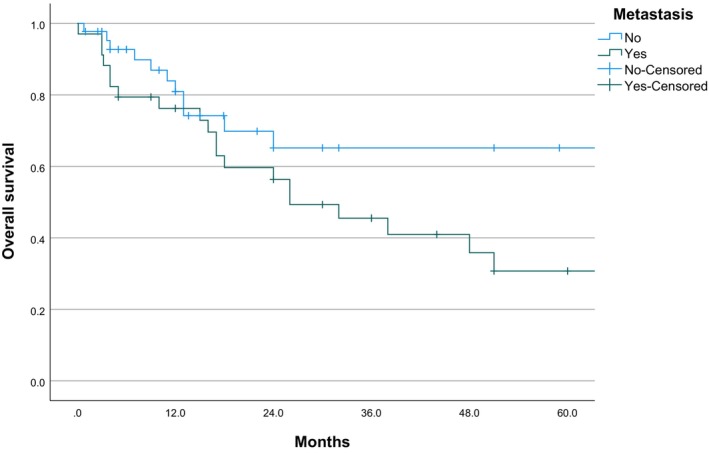
Survival curve (1).

**FIGURE 4:**
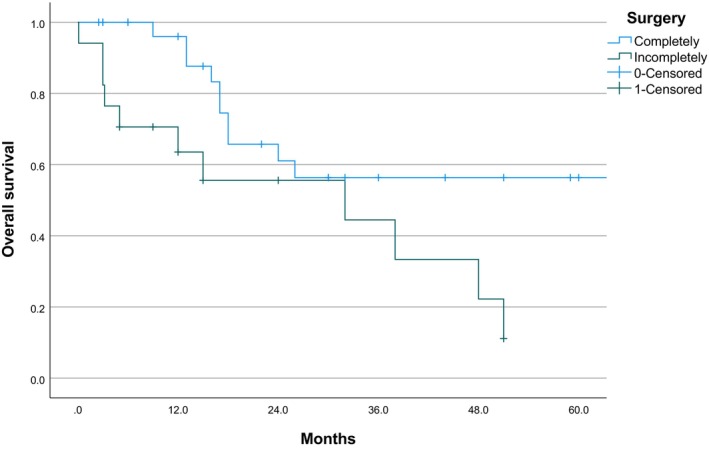
Survival curve (2).

According to the results of Kaplan–Meier univariate analysis and Log‐rank test, the presence of lymph node and/or distant metastasis (*χ* ^2^ = 3.996, *p* = 0.046) and incomplete surgery (*χ* ^2^ = 5.586, *p* = 0.018) were identified as the main prognostic factors for this disease. Among the 44 metastasis‐free patients, 13 (29.55%) died, with a mean survival period of 151.774 months (95% CI: 58.744–244.804) and a 5‐year survival rate of 65%. In contrast, among the 34 patients with metastasis, 21 individuals (61.76%) had a fatal outcome, with an average survival period of 47.695 months (95% CI: 28.939–66.451) and a median survival time of only 26 months (95% CI: 2.280–93.720), with a 5‐year survival rate of 31%. Statistically, the 5‐year survival rate for cases with metastasis ending in death was only 4.76%, and the 2‐year survival rate was 38.10%. Furthermore, relative to patients with complete surgical resection, there were 10 deaths (33.33%), with a mean survival of 81.941 months (95% CI: 58.482–105.400). In contrast, 11 of the 17 patients with incomplete surgery (64.71%) ultimately died, with a significantly lower average survival of 27.338 months (95% CI: 16.733–37.943) and a 5‐year survival rate of 0% (Table 6; Figures 3 and 4). The data indicate that prognosis is not significantly correlated with the following clinically relevant features: age, sex, tumor marker, site/size of tumor, compression growth, obstruction of bile/pancreatic duct, and the use of adjuvant therapy (*p* > 0.05).

**TABLE 6:** Kaplan–Meier method of univariate survival analysis^a^.

Item	*n*	Average ST(M) (95% CI)	Median ST(M) (95% CI)	*χ* ^2^	*p*
Age (Y)					
<45	42	130.806 (67.199–194.413)	32.000 (6.428–57.572)	0.508	0.476
≥45	36	80.078 (54.013–106.143)	85.000 (0–174.511)		
Sex					
Men	42	45.780 (33.389–58.171)	48.000 (8.373–87.627)	0.983	0.321
Women	36	135.938 (50.094–221.781)	143.7 (0–288.023)		
Metastasis					
No	44	151.774 (58.744–244.804)	143.700 (41.420–245.980)	3.996	0.046
Yes	34	47.695 (28.939–66.451)	26.000 (2.280–93.720)		
Tumor marker					
No	24	48.806 (28.140–69.473)	26.000 (22.275–29.725)	0.718	0.397
Yes	18	26.804 (15.963–37.645)	18.000 (7.095–28.905)		
Site					
Head	29	101.670 (37.016–166.323)	51.000 (8.459–93.541)	3.750	0.290
Body	5	20.440 (5.241–35.639)	32.000 (–)		
Tail	27	59.167 (39.562–78.772)	–		
Other	6	29.600 (2.802–56.398)	18.000 (6.385–29.615)		
Size					
<10 cm	40	65.831 (42.526–89.136)	48.000 (20.179–75.821)	2.056	0.152
≥10 cm	19	212.960 (111.660–314.260)	–		
Compression growth					
No	51	116.158 (47.059–185.256)	72.200 (26.364–118.036)	0.119	0.730
Yes	16	65.229 (32.153–98.304)	32.000 (20.567–43.433)		
Obstruction					
No	57	66.587 (46.206–86.968)	38.000 (4.194–71.806)	1.922	0.166
Yes	10	222.921 (110.314–335.527)	–		
Surgery^b^					
Complete	30	81.941 (58.482–105.400)	–	5.586	0.018
Incomplete	17	27.338 (16.733–37.943)	32.000 (0–74.874)		
Adjuvant therapy					
No	34	73.141 (47.359–98.924)	48.000 (–)	0.433	0.511
Yes	32	54.507 (33.053–75.961)	26.000 (8.425–43.575)		

^a^ ST, survival time; M, month; Y, years old.

^b^ Complete, refers to radical resection of pancreatic tumors; Incomplete, refers to the absence of radical surgery (including palliative surgery).

## 4 DISCUSSION

Adult primary PB is an aggressive malignant tumor that, despite its clinical rarity, can be fatal. Current research on PB predominantly comprises case reports, with limited comprehensive analyses and studies on factors influencing prognosis. This article aims to fill this gap by conducting a thorough analysis.

This study demonstrates that PB is not only common in infants and adolescents but also occurs in adults, affecting a wide age range. Notably, the proportion of young adults (<45 years) affected by this disease is higher than in other age groups, indicating a predilection for younger individuals even in adulthood. Additionally, PB shows a low gender preference, which is consistent with previous literature.^1^ The pancreas is deeply located behind the peritoneum, making the onset of PB insidious and difficult to detect clinically. The clinical manifestations of the disease lack specificity but are diverse. Some patients seek medical attention due to incidental discovery or examination revealing abdominal masses, with early‐stage cases often being asymptomatic. As the tumor volume increases, its growth infiltrates or compresses surrounding tissues, invading the peritoneum and causing local pain. This study indicates that nearly half of PB patients present with abdominal pain as their primary complaint at the initial consultation. Weight loss is the second most common symptom, likely related to the large amount of energy and nutrients consumed by the body during the growth of malignant tumor cells. In addition, at the time of initial diagnosis, lymph node or distant metastases occurred in 40.78% of patients, the latter being predominantly metastatic to a single distant organ, most commonly the liver. Consequently, PB patients may exhibit symptoms of jaundice or impaired liver function due to liver involvement. A minority of patients may also develop ascites. Lymph node and lung metastases follow liver metastasis in frequency.

Laboratory results have low specificity and limited clinical diagnostic significance. Some literature suggests that AFP has considerable value in the diagnosis and detection of PB in children.^1^ However, we found that only a small subset of adult PB cases exhibit elevated tumor markers, including AFP and CA19‐9, which may be instructive, and vigilance is still needed when the condition is clinically present. Liver enzyme elevation may occur when PB invades or metastasizes to the liver, affecting liver function. Due to the insidious nature of PB, its diagnosis often requires imaging studies for assistance. Most primary lesions involve the pancreatic head, typically large, with a maximum diameter of up to 21.5 cm, usually appearing as a round‐like mass with well‐defined borders due to the presence of the envelope. The lump can be solid or cystic‐solid and may be accompanied by calcifications or necrosis, showing uneven mild to moderate enhancement on contrast‐enhanced imaging and rapid enhancement of the capsule. Besides encroaching upon surrounding blood vessels, the tumor mass may compress and displace adjacent tissues and organs, commonly invading the duodenum, stomach, and small intestine. When located in the pancreatic head, bile duct dilation may be observed. Among the cases included in this study, only a small subset provided relevant MRI and ultrasound findings, precluding comprehensive statistical analysis. However, these examinations have clinical implications. Literature^25^ suggests that PB manifests mainly as low to moderate signal intensity on T1‐weighted imaging (T1WI), similar to the signal intensity of the spleen, and slightly high or high signal intensity on T2‐weighted imaging (T2WI). Ultrasound typically shows cystic‐solid hypoechoic masses. These imaging findings are consistent with those observed in pediatric populations with PB.^58^

The clinical features and imaging results of PB lack specificity, and the final diagnosis still relies on pathological findings. The tumor exhibits abundant epithelial and stromal components, usually distributed in a lobular pattern, with these lobules separated by dense fibrous septa. The epithelial components tend to undergo multidirectional differentiation, leading to heterogeneity. Acinar differentiation is common in PB. Though in this study only 40% of cases definitively mentioned acinar differentiation, this may be due to variability in reporting of pathologic features. The nuclei of the tumor cells are partially prominent and display nuclear heterogeneity. Squamous corpuscles are characteristic pathological changes of PB, typically showing low differentiation, which is crucial for establishing the diagnosis. When histological features are atypical, diagnosis requires the integration of IHC results. Significant staining for Trypsin, Chymotrypsin and BCL‐10 is typical IHC evidence of acinar differentiation, which is the common line of differentiation in PB. Additionally, abnormal expression of β‐catenin in the nucleus and cytoplasm is commonly observed in squamous corpuscles, believed to be due to a point mutation of the β‐catenin gene, leading to the overexpression of cyclin D1.

The etiology and pathogenesis of PB remain unclear, but in recent years, the pathogenic molecular mechanisms have been gradually analyzed. Studies^68^, ^69^ have shown a close association between childhood PB and FAP/Beckwith–Wiedemann syndrome (BWS), while adult PB seems to have no direct relationship with the diseases mentioned above. In this study, only three cases had a confirmed history of FAP. However, from a molecular perspective, among cases with reported gene mutations, 71.43% of patients experienced alterations in the APC/β‐Catenin pathway, related to mutations in the APC and CTNNB1 genes. Aberrant activation of this pathway may affect WNT signal transduction pathways and participate in the proliferation, invasion, and metastasis of tumor cells. Abraham et al.^69^ reported that loss of heterozygosity of the 11p chromosome locus is also a common genetic alteration in PB. This phenomenon can also occur in hepatoblastoma and Wilms tumor, suggesting that frequent loss of heterozygosity of the 11p locus may be a common genetic pathway in embryonic tumors. However, this is not prominently evident in adult PB in this study. Reissig et al.^70^ confirmed the possibility of amplification of MCL1 in adult patients with PB in a study, suggesting that such patients may benefit from MCL1‐targeted inhibition. As molecular mechanisms continue to be elucidated, identifying genetic biomarkers may facilitate the discovery of novel targeted therapeutic strategies. Research on targeted drugs for CTNNB1 mutations is continually evolving, yet no specific drugs have been widely adopted for clinical use. Although the multi‐target drug Cabozantinib has shown some efficacy in endometrial cancer patients, its effectiveness in PB remains unproven.^71^ Tegavivint is an innovative drug that selectively inhibits the Wnt/β‐catenin signaling pathway. By targeting Transducin Beta‐like Protein 1 (TBL1), it effectively suppresses the oncogenic activity of β‐catenin, inhibits tumor growth, and enhances T‐cell infiltration, as demonstrated in animal models of hepatocellular carcinoma.^72^ Ongoing clinical projects are investigating the potential efficacy of Tegavivint across various types of cancer, raising hopes for its potential efficacy in PB. We found that a few adult PB cases also exhibit mutations in the SMAD4, MEN1, RB1, and TSC2 genes, or FGFR2‐INA fusion, sometimes combining two mutations. These are all potential therapeutic targets for PB. Besides, unlike pancreatic ductal adenocarcinoma, KRAS gene mutations or abnormal expression of P53 were not found in adult patients of PB. Molecular targeted research for PB is in its nascent stages, requiring more foundational studies and clinical trials for drug development for clinical use.

Several cases of PB being misdiagnosed were found in this study. The most common misdiagnosis is PanNENs, which are similar to PB due to their structural diversity and frequent expression of neuroendocrine markers. However, PanNENs differ in having nuclei with a more pronounced salt‐and‐pepper chromatin appearance.^73^ PanNENs also commonly show strong expression of Syn, CgA, and CD56, weak expression of pancreatic enzymes, and may exhibit hormone activity (insulin, glucagon, etc.), but lack squamous corpuscles. Solid pseudopapillary neoplasm of the pancreas is frequently seen in young women.^74^ Microscopically, it presents rich solid nests of cells, which may be eosinophilic or vacuolated. Foam cells can be observed between cells, and nuclear grooves are visible, while acinar or ductal differentiation and squamous corpuscles are absent. It has a better prognosis compared to PB.^75^ PB with predominantly acinar differentiation is difficult to distinguish from pancreatic acinar cell carcinoma. Both can show acinar, neuroendocrine, and ductal differentiation and usually exhibit immunoreactivity for pancreatic enzymes and BCL‐10. However, pancreatic acinar cell carcinoma is more common in the elderly,^76^ often presents with metastasis at the time of diagnosis, and has an inferior prognosis. Squamous corpuscles are key distinguishing features, and positive β‐catenin immunohistochemistry results help in differential diagnosis. Recent study^77^ has shown that DNA methylation profiling can be a valuable auxiliary method to differentiate the diagnosis of various pancreatic neoplasms, minimizing the risk of misdiagnosis. We expect that this technology may be used in the clinic practice in the future. In conclusion, clinicians should be vigilant when encountering patients with abdominal pain, weight loss, or an abdominal mass, and should not exclude the possibility of PB, especially in younger individuals. The definitive diagnosis of this disease relies on pathological examination, with primary features being acinar differentiation and prominent squamous corpuscles.

There are no defined treatment guidelines for PB. Based on the results of this study, we recommend that complete surgical resection be the first option, if available. When adjacent organs are involved, they should be resected along with the affected tissue. Complete excision is advantageous for long‐term survival, compared to patients who cannot undergo complete surgical excision and have a 5‐year survival rate of 0%. In addition, chemotherapy and radiotherapy are also optional treatments. Some authors recommend chemotherapy exclusively for patients with recurrent, residual, or unresectable PB.^53^ However, given the malignancy of this disease, most scholars advocate for the aggressive use of adjunctive therapy in all cases.^31^ In the study, we found that chemotherapy, either alone or in combination with surgery, is often utilized in the treatment of PB, with varying outcomes. Combination chemotherapy with platinum‐based drugs and ADM is the most common regimen and serves as the primary chemotherapeutic agent for PB currently. In posterior line therapy, 5‐FU‐based regimens such as FOLFIRINOX are commonly used. Currently, clinical treatment can be based on these empirical chemotherapy regimens. Radiotherapy is typically administered after or in combination with chemotherapy. The efficacy of chemotherapy and radiotherapy remains to be determined and seemingly lacks a significant impact on prognosis. However, there still need to be more PB cases analyzed, which may affect the conclusions. Immunotherapy and targeted therapy can be employed in PB treatment, although protocols are not standardized and currently serve as tentative options. Prognosis is relatively favorable for PB patients without metastasis, but the presence of lymph nodes and/or distant metastases significantly impacts prognosis, with a median survival of only 26 months and an average survival of 48 months. Despite some adult PB cases exhibiting long‐term survival, the prognosis remains inferior to pediatric PB,^74^ possibly due to the ease of early detection of abdominal masses in children.

Rare tumors present clinical challenges. Initially, it was widely believed that PB occurred only in children, but it is now clear that no gender or age is excluded. When rapid abdominal mass enlargement accompanied by abdominal pain occurs, even if the likelihood is low, the possibility of PB should be considered, prompting comprehensive examinations. To date, there are no large cohorts of adult patients with PB, only relatively scarce cases, leading to potential limitations in analysis and the results of the survival analyses may be biased by the heterogeneity of each case report. It is recommended that PB be clinically emphasized, with long‐term follow‐up of PB patients to further research this disease.

## 5 CONCLUSION

As a rare malignant tumor, adult PB lacks specific clinical and radiological characteristics. Diagnosis primarily relies on histopathology and IHC findings, with particular emphasis on identifying unique features, including squamous corpuscles, acinar differentiation, and the expression of acinar markers. These features need to be carefully reviewed by pathologists to prevent misdiagnosis. Surgery is the primary treatment method for PB. Due to its invasive nature, the prognosis is poor if surgical intervention is missed or if complete resection is not possible at the time of diagnosis with metastases already present.

## AUTHOR CONTRIBUTIONS

**Jiaqian Yuan:** Conceptualization (lead); data curation (lead); methodology (lead); validation (lead); visualization (lead); writing – original draft (lead). **Yong Guo:** Supervision (equal); writing – review and editing (equal). **Yan Li:** Supervision (equal); writing – review and editing (equal).

## FUNDING INFORMATION

This research is supported by the Research on Machine Learning Model for Predicting the Effect of Spleen Strengthening and Qi‐Regulating Therapy of TCM on Recurrence and Metastasis of breast cancer Based on Dynamic Changes of System Immunity(Grant No. 2024ZL382); the National Famous Traditional Chinese Medicine Expert Inheritance Studio; the National Natural Science Foundation of China (Grant No. 81973805); Zhejiang Provincial TCM Science and Technology Project (Grant No. 2015ZA088); Zhejiang Provincial Project for the key discipline of traditional Chinese Medicine (Yong GUO, no. 2017‐XK‐A09, http://www. zjwjw.gov.cn/).

## CONFLICT OF INTEREST STATEMENT

The authors declare no competing financial interest.

## Data Availability

All data generated or analyzed during this study are included in this published article.
