# Correction to “Diagnosis, Treatment, and Prognosis of Adult Pancreatoblastoma”

**DOI:** 10.1002/cam4.72345

**Published:** 2026-09-28

J. Yuan, Y. Guo, and Y. Li. “Diagnosis, Treatment, and Prognosis of Adult Pancreatoblastoma,” *Cancer Medicine* 13, no. 16 (2024): e70132, https://doi.org/10.1002/cam4.70132.

Author Yan Li was incorrectly associated with affiliation “The First Clinical Medical College, Zhejiang Chinese Medical University, Hangzhou, China; Department of Medical Oncology”; however, the correct affiliation for Yan Li is “Department of Medical Oncology, The First Affiliated Hospital of Zhejiang Chinese Medical University, Hangzhou, China” only.

The online version of this article has been corrected accordingly.

We apologize for this error.

